# Nasal and maxillary sinus volume change in patients with obstructive sleep apnea after bimaxillary advancement surgery

**DOI:** 10.1186/s12903-023-03657-w

**Published:** 2023-11-17

**Authors:** Georgia Tzironi, Álvaro Zubizarreta-Macho, Joan Brunsó-Casellas, Valentín Cabriada-Nuño, Ana Belén Lobo Galindo, Alberto Albaladejo Martínez, Félix De Carlos-Villafranca

**Affiliations:** 1 Department of Surgery, Faculty of Medicine, University of Salamanca, 37008 Salamanca, Spain; 2 Department of Endodontics, Faculty of Health Sciences, Alfonso X El Sabio University, 28691 Madrid, Spain; 3 Department of Implant Surgery, Faculty of Health Sciences, Alfonso X El Sabio University, Avda. Universidad, 1. 28691. Villanueva de La Cañada, Madrid, Spain; 4 Maxillofacial Surgeon, Cruces University Hospital, Barakaldo, Spain; 5 Sleep and Ventilation Unit, Respiratory Division, Cruces University Hospital, Barakaldo, Spain; 6 Department of Surgery and Medical-Surgical Specialties, Area of Orthodontics, Faculty of Medicine, University of Oviedo, Oviedo, Spain

**Keywords:** Orthodontics, Bimaxillary advancement surgery, Maxillary sinus, Nasal airway, Obstructive sleep apnea

## Abstract

The airway complex is modified by bimaxillary advancement surgery performed in patients suffering from obstructive sleep apnea (OSA). The aim of the present study is to analyse the volume of nasal and maxillary sinus after bimaxillary advancement surgery in patients suffering from OSA. The maxillary sinus and nasal complex of eighteen patients with OSA was measured through cone-beam computed tomography (CBCT) before and after they were treated with bimaxillary advancement surgery. Digital planning software was used to effectively measure the upper volume changes, as well as, statistical analysis of the results was performed.

**Methods **Eighteen patients were diagnosed with OSA the severity of which was measured by the apnea hypopnea index and were selected and submitted to preoperative and postoperative CBCT scans. Afterwards, datasets were uploaded into therapeutic digital planning software (Dolphin Imaging) to measure the volume of the right and left maxillary sinus and nasal and maxillary sinus complex. Statistically analysis between preoperative and postoperative measures was performed by Student *t*-test statistical analysis.

**Results **The paired *t*-test showed statistically significant volumetric reductions in the left maxillary sinus (*p* = 0.0004), right maxillary sinus (*p* < 0.0001) and nasal and maxillary sinus complex (*p* = 0.0009) after bimaxillary advancement surgery performed in patients suffering from OSA.

**Conclusion** The results showed that bimaxillary advancement surgery reduces the maxillary sinus volume as well as, the fossa nasal and sinus complex volume.

## Background

Obstructive sleep apnea (OSA) is a disorder that affects between 2%-4% of the adult population; consequently, the public attention to this condition has increased over the past few years [1]. The main sign is the repetitive collapse of the upper airway during sleep, and it is associated with lack of oxygen in blood [2]. A great number of other symptoms may accompany this disorder, such as, sleepiness, lack of concentration due to bad quality of sleep, snoring, interrupted breathing, gasping or chocking episodes during sleep etc., which may lead the patient to the search for treatment [1].

The usual diagnostic procedure in order to verify the presence and detect the severity of OSA includes a testing called polysomnography (PSG). This is a method that measures neurological and cardio-respiration parameters through sensors so that it can detect ventilation cease, and arousal due to airway obstruction [2]. Sleep-disordered breathing is measured by the apnea hypopnea index (AHI) that takes into account the number of apnea and hypopnea episodes that occur during sleep, divided by the hours of sleep [3]. The American Academy of Sleep Medicine classifies the severity of OSA accordingly: Between 5 and 15 episodes per hour is considered a mild situation, from 16 to 30 is classified as moderate, and more than that is characterized as severe [1, 3]. Additionally, OSA can be classified according to the period of duration. In cases where the episodes last less than two weeks, it can be characterized as acute, subacute if it lasts between 2 weeks and 6 months and chronic for 6 months or longer [4]. Moreover, most patients begin to demonstrate symptoms between the ages of 40 and 60 [5] and the prevalence is double in men compared with women [6]. Predisposing factors may include obesity especially in the cervical area [7], Cushing ‘s syndrome [8], retroglossal space volume, as well as neck size [8, 9]. Other factors such as, hyoid position, soft palate length etc., may also predispose to OSA even though their influence may be smaller than the factors mentioned above [7]. The need of treatment is constantly increasing as new studies have been able to highlight the relationship between OSA and cardiovascular episodes, such as, hypertension, coronary artery disease, stroke and heart failure [10].

In adults the treatment options include non-invasive procedures such as, weight loss, behavioural modification, medication, continuous positive airway pressure (C-pap), oral appliance therapy etc., and invasive procedures that involve surgical methods such as maxillomandibular advancement, surgically assisted rapid maxillary expansion, uvulopalatoplasty, hypoglossal nerve stimulation etc. [11]. Although a more invasive procedure is less often preferred by both patients and clinicians, a meta-analysis conducted in 2016 showed encouraging results for the treatment of OSA after maxillomandibular advancement surgery [12].

The aim of the present study is to analyse the volume of nasal and maxillary sinus after bimaxillary advancement surgery in patients suffering from OSA, with a null hypothesis (H0) stating that there will be no differences between sinuses and nasal airway volume, before and after maxillomandibular advancement surgery performed in patients suffering from OSA.

## Methods

### Study design

The present study was performed at the Department of Orthodontics of the University of Salamanca (Salamanca, Spain) and the Department of Orthodontics of the University of Oviedo (Oviedo, Spain), between April 2021 and May 2022, in accordance with the ethical guidelines established by the Declaration of Helsinki and was authorized by the Ethical Committee of the Faculty of Medicine of the University of Oviedo (Oviedo, Spain), in March 2021 (process CEIC E19/39). The patients gave their consent to provide the preoperative and postoperative CBCT scans.

### Experimental procedure

Eighteen white Caucasian male patients between 32 and 52 years old with moderate-severe OSA in non-specific or supine position, indication determined by a sleep pathology committee (pneumology, otorynolaryngology, maxillofacial, dentistry, radiology, and neurophysiology) were consecutively selected.

**Fig. 1:**
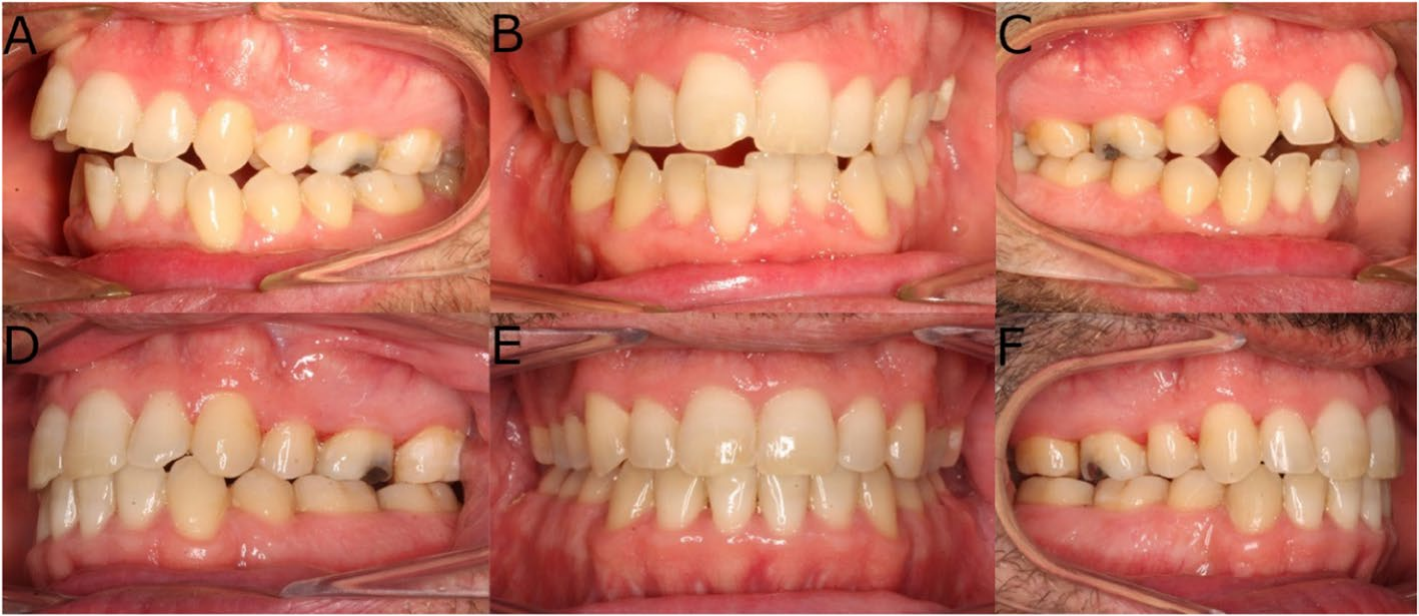
**A** Preoperative intraoral images of left lateral, (**B**) front and (**C**) right lateral view and (**D**) postoperative intraoral images of left lateral, (**E**) front and (**F**) right lateral view

Exclusion criteria were applied as follows: pregnant woman, predominant apneas of central origin, major craniofacial deformities (such as cleft palate, severe asymmetric malocclusion, trauma or head & neck oncologic surgery), and different surgery than maxillomandibular advancement. The patients selected were submitted to Lefort I maxillary osteotomy, Obwegeser-Dal Pont mandibular split, and movements pivoted on the upper central incisor (UCI) are planned in three-dimension (3D). The goal is to provide to the patient the most stable occlusion, which will be the same as the initial one if pre-operative orthodontics has not been implemented. Genioplasties are also considered in order to provide optimal profile aesthetics. Planned surgical movements are 10.40 mm of advancement and 2.11 of maxillary anterior impaction. In this way counterclock mandibular rotation is achieved (Fig. 1). Informed consent was obtained from all parents.

### Measurement procedure

**Fig. 2:**
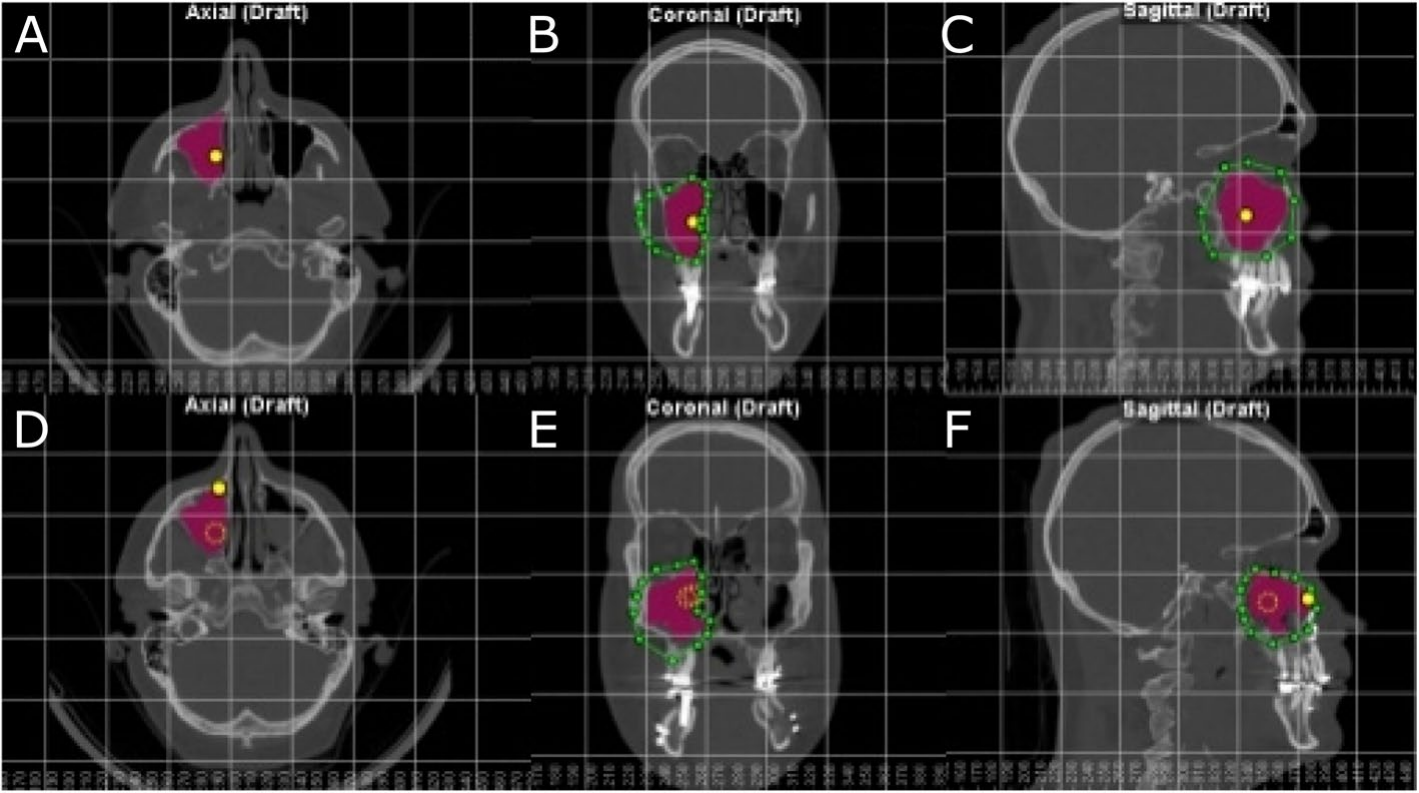
**A** Axial, (**B**) coronal and (**C**) sagittal plane of the preoperative CBCT scans and (**D**) axial, (**E**) coronal and (**F**) sagittal plane of the postoperative CBCT scans. Green line describes the selected area, yellow points define the air density and purple area describes the volume of the left maxillary sinus

All patients were submitted to preoperative cone-beam computed tomography (CBCT) scan (WhiteFox, Satelec, Merignac, France) with the following exposure parameters: 105.0 kV peak, 8.0 mA, 7.20 s, and a field of view of 15 mm × 13 mm, by aligning the Frankfort plane to the floor with frontal and chin support and a postoperative CBCT scan 6 months after performing bimaxillary advancement surgery, to prevent radiological distortion due the tissue inflammation. Afterwards, the preoperative and postoperative CBCT scans (WhiteFox, Satelec, Merignac, France) were uploaded into therapeutic digital planning software (Dolphin Imaging, Dolphin Imaging & Management Solutions, Chatsworth, CA, USA) to allow the accurate measurement of the volume of the right maxillary sinus, left maxillary sinus and nasal and maxillary sinus complex. The volumes were measured after selecting the anatomical area in the axial, coronal and sagittal plane and ensuring the air density measurement by reference points placement inside the selected area. In every plane it was chosen the most extreme point and through those points a drawing was made so that it included the area chosen for measurement. Then, through the program the appropriate sensitivity was selected so that all the airway points within the selected field were included (Fig. 2). Finally, each drawing was measured digitally. It is important to mention that in each scan, the same sensitivity was used for all the airway measurements.

**Fig. 3:**
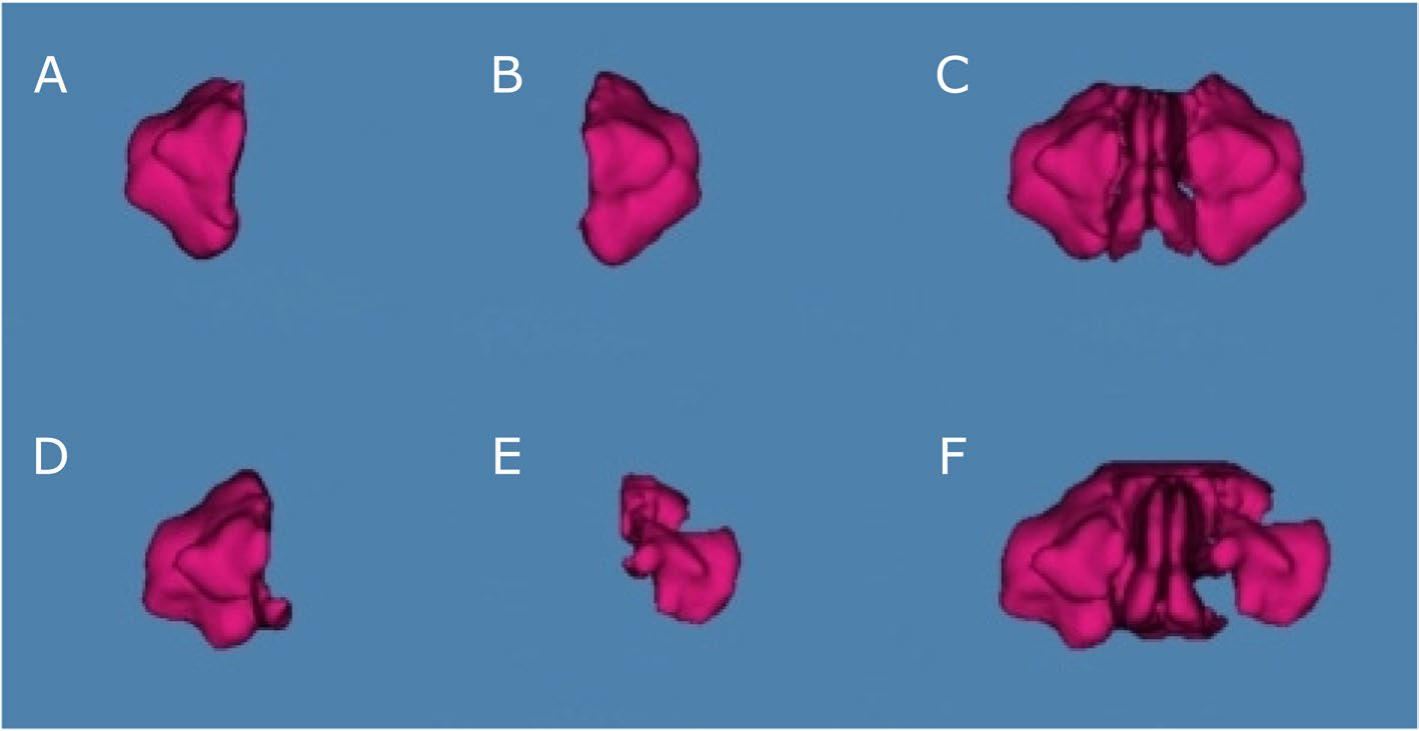
**A** Preoperative assessment of the right maxillary sinus, (**B**) left maxillary sinus and (**C**) nasal and maxillary sinus complex and (**D**) postoperative assessment of the right maxillary sinus, (**E**) left maxillary sinus and (**F**) nasal and maxillary sinus complex

Subsequently, the therapeutic digital planning software (Dolphin Imaging, Dolphin Imaging & Management Solutions, Chatsworth, CA, USA) allowed the accurate measurement of the volume of the right maxillary sinus, left maxillary sinus and nasal and maxillary sinus airway complex after performing bimaxillary advancement surgery using the Airway Measurement tool (Fig. 3). All measurement procedures were performed by a unique operator with prior experience. All experimental procedures were performed, according to a previous study, where the authors demonstrated that this digital technique is a repeatable, reproducible, and acute measurement technique for analyzing the volume of the nasal and maxillary sinus after suture palatine expansion using the Hyrax disyuntor. The repeatability and reproducibility of these digital measurements were analyzed using Gage R&R statistical analysis to validate the digital measurement technique [13].

### Statistical tests

Statistical analysis was conducted by SAS v9.4 (SAS Institute Inc., Cary, NC, USA). Descriptive analysis was expressed as means and standard deviations (SD). Shapiro–Wilk normality test was performed. Comparative analysis of the right maxillary sinus (mm^3^) left maxillary sinus (mm^3^) and nasal and maxillary sinus complex (mm^3^) after performing bimaxillary advancement surgery was performed using Student’s t-test. The statistical significance was set at *p* < 0.05.

## Results

Demographic data of the patients enrolled in the study is presented in Table 1.

**Table 1:** Demographic data of the patients enrolled in the study

	Age	BMI	ADV_SCI	VAM_SCI	VPM_1_6	VPM_2_6	ADV
Mean	40.2	25.7	10.4	-2.10	1.94	1.94	13.9
SD	5.67	3.35	0.530	1.80	1.18	1.13	1.85
Minimum	32	21.3	9.70	-7.00	0.00	0.00	11.3
Maximum	50	33.0	11.0	-1.00	4.10	3.90	17.0

*BMI* body mass index, *SCI* superior central incisor, *ADV* advancement, *VAM* vertical anterior movement, *VPM* vertical posterior movement

**Fig. 4:**
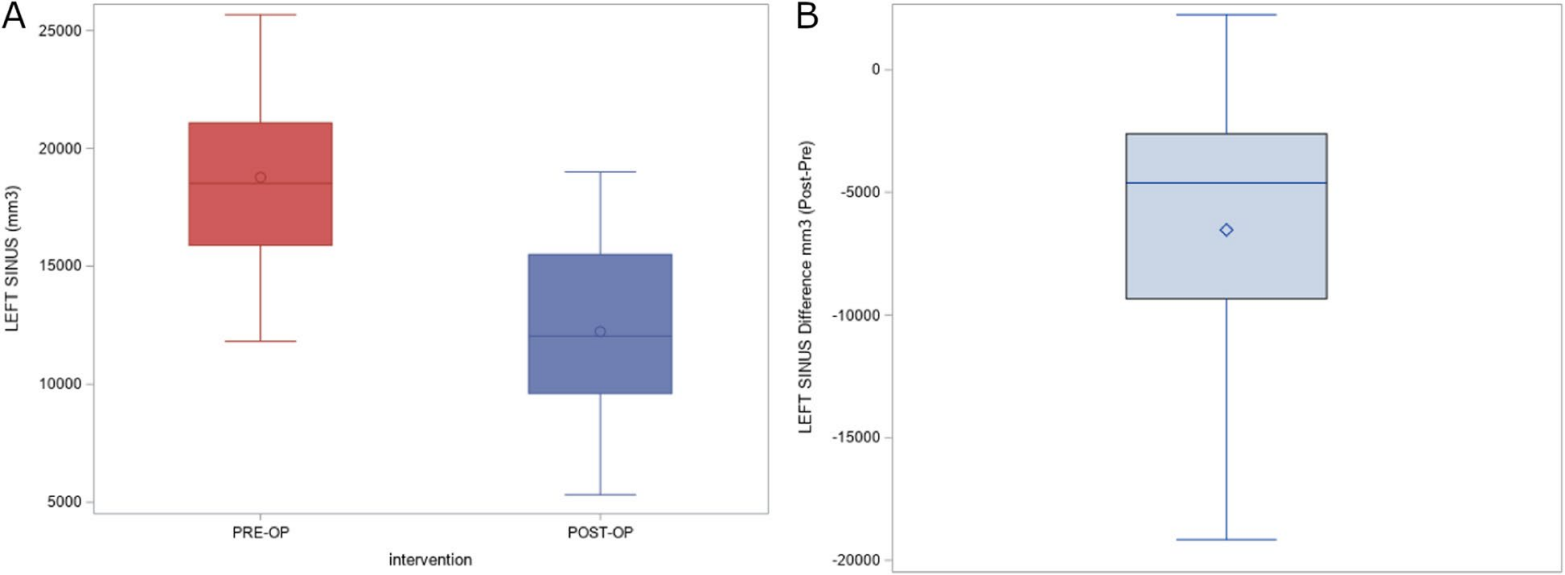
**A** Box plot of the preoperative and postoperative measurement volumes of the left maxillary sinus and the (**B**) volumetric differences between them. The horizontal line in each box represents the respective median value of the study groups. ◊, o, Mean value of the box plots

The means and SD values for the preoperative and postoperative volumes of the left maxillary sinus (mm^3^) after performing bimaxillary advancement surgery are displayed in Table 2; moreover, volume differences of the left maxillary sinus (mm^3^) after performing bimaxillary advancement surgery are also displayed in Table 2. The paired *t*-test analysis showed statistically significant differences between the preoperative and postoperative volumes of the left maxillary sinus (mm^3^) after performing bimaxillary advancement surgery (*p* = 0.0004) (Fig. 4).

**Table 2:** Descriptive statistics of the preoperative and postoperative volumes of the left maxillary sinus (mm^3^), right maxillary sinus (mm^3^), nasal and maxillary sinus complex (mm^3^) and the volumetric differences of the nasal and maxillary sinus complex (mm^3^) after performing bimaxillary advancement surgery

	n	Mean	SD	Min	Max
Left maxilary sinus (mm^3^)					
Pre-operative	18	18,771.83	3895.13	11,823.00	25,672.00
Post-operative	18	12,238.56	4047.63	5314.00	18,996.00
Volumetric differences	18	-6533.3	6339.4	-19,152	2248
Right maxilary sinus (mm^3^)					
Pre-operative	18	20,183.50	6622.13	11,550.00	36,472.00
Post-operative	18	12,664.33	5285.14	328.00	20,049.00
Volumetric differences	18	-7519.2	6705.3	-22,231	1443
Nasal and maxilary sinus (mm^3^)					
Pre-operative	18	62,008.94	10,268.09	40,056.00	74,918.00
Post-operative	18	50,745.22	8979.96	38,953.00	68,721.00
Volumetric differences	18	-11,263.7	11,874.3	-33,057	4448

**Fig. 5:**
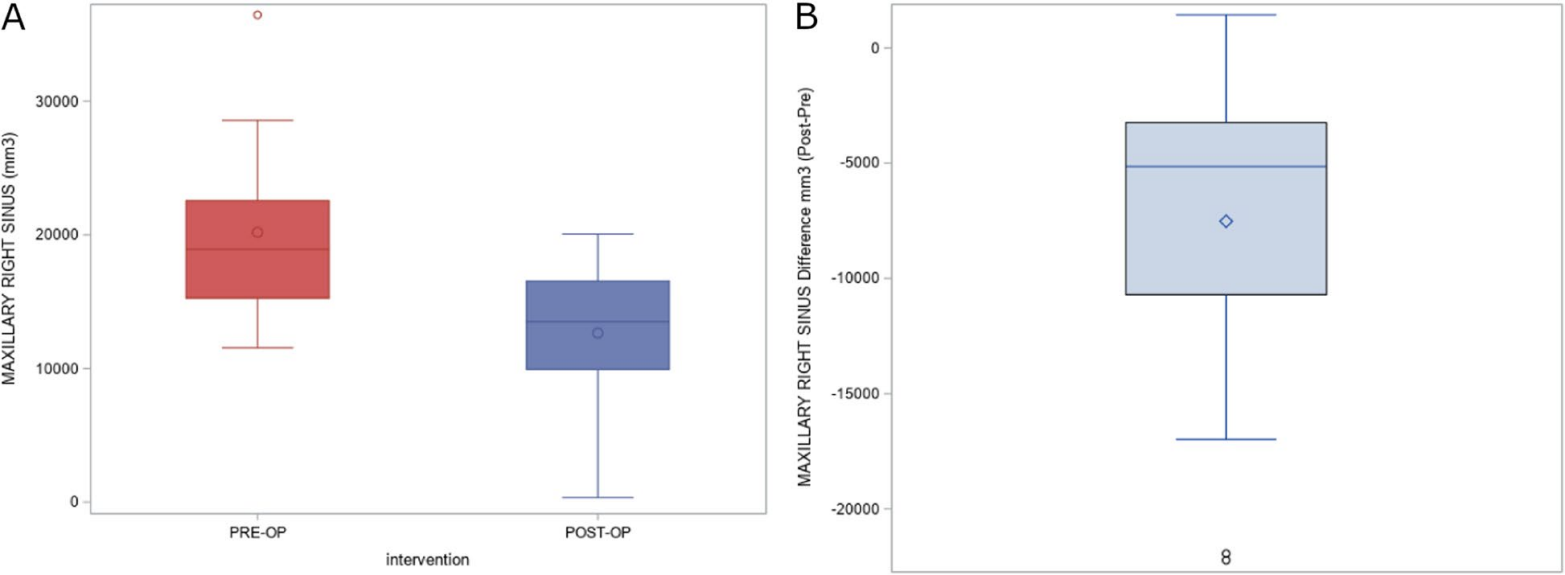
**A** Box plot of the preoperative and postoperative measurement volumes of the left maxillary sinus and the (**B**) volumetric differences between them. The horizontal line in each box represents the respective median value of the study groups. ◊, o, Mean value of the box plots

The means and SD values for the preoperative and postoperative volumes of the right maxillary sinus (mm^3^) after performing bimaxillary advancement surgery are displayed in Table 2; moreover, volume differences of the right maxillary sinus (mm^3^) after performing bimaxillary advancement surgery are also displayed in Table 2. The paired *t*-test analysis showed statistically significant differences between the preoperative and postoperative volumes of the right maxillary sinus (mm^3^) after performing bimaxillary advancement surgery (*p* < 0.0001) (Fig. 5).

**Fig. 6:**
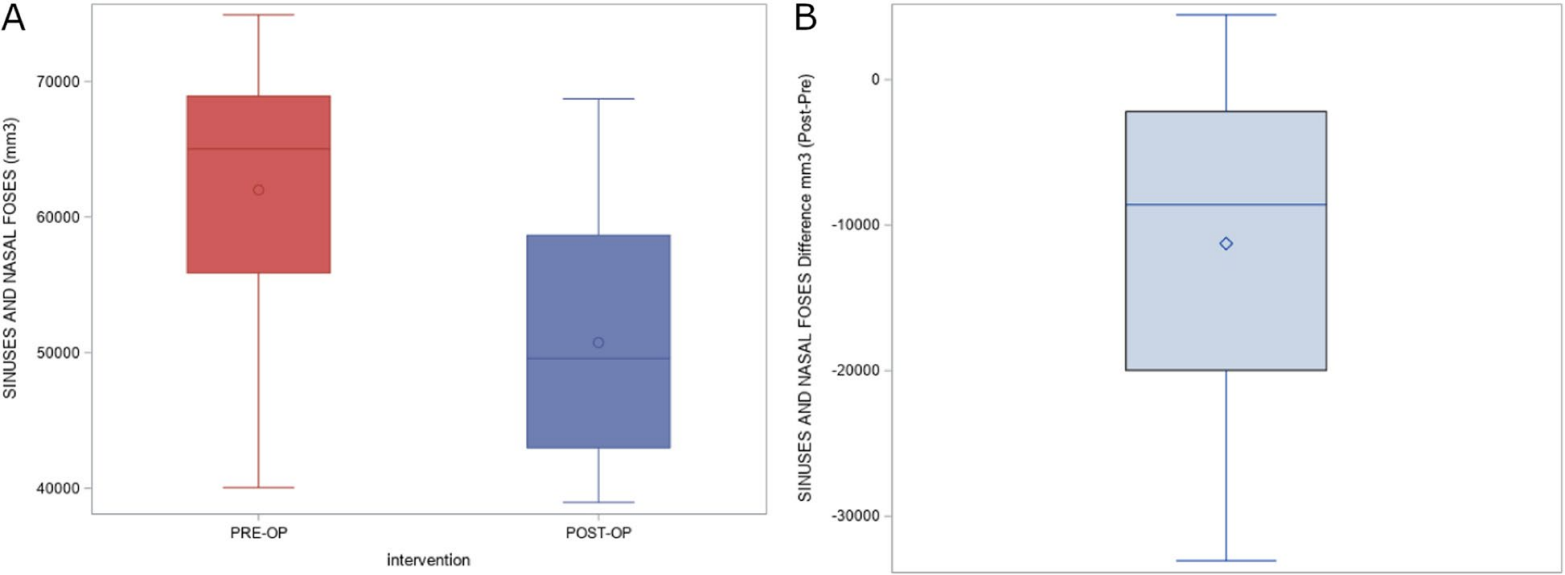
**A** Box plot of the preoperative and postoperative measurement volumes of the nasal and maxillary sinus complex and the (**B**) volumetric differences between them. The horizontal line in each box represents the respective median value of the study groups. ◊, o, Mean value of the box plots

The means and SD values for the preoperative and postoperative volumes of the nasal and maxillary sinus complex (mm^3^) after performing bimaxillary advancement surgery are displayed in Table 2; moreover, volume differences of the nasal and maxillary sinus complex (mm^3^) after performing bimaxillary advancement surgery are also displayed in Table 2. The paired *t*-test analysis showed statistically significant differences between the preoperative and postoperative volumes of nasal and maxillary sinus complex (mm^3^) after performing bimaxillary advancement surgery (*p* = 0.0009) (Fig. 6).

Apnea/Hypopnea index was measured by polysomnography (Aura Grass, Astro-Med Industrial Park, 600 East Greenwich Avenue West Warwick, RI 02893 U.S.A.) was found to be reduced by a median of 30.50 points after surgery and an average of 80% of patients was found to have an AHI under 15 points (Table 3). In resume, the right maxillary sinus showed a higher volume decrease after performing bimaxillary advancement surgery, comparing to the left maxillary sinus and the nasal cavity sinus complex showed the highest decrease overall. Thus, the bimaxillary advancement surgery might lead an asymmetric volume change in the maxillary sinus but also to nasal volume increase.

**Table 3:** Pre and postoperative values of apnea/hypopnea index using polysomnography

Patient	AHI	AHI Sup	AHI NSup	PSO2 BAS (%)	PSO2 MIN (%)	Patient	AHI	AHI Sup	AHI NSup	PSO2 BAS (%)
Patient	PRE	POST	PRE	POST	PRE		PRE	POST	PRE	POST
01	37	23	37	41	37	01	37	23	37	41
02	68	5	67	7	73	02	68	5	67	7
03	31	7	59	12	11	03	31	7	59	12
04	33	0	48	0	31	04	33	0	48	0
05	29	2	50	2	9	05	29	2	50	2
06	35	9	35	14	-	06	35	9	35	14
07	61	16	61	16	-	07	61	16	61	16
08	85	8	88	8	69	08	85	8	88	8
09	76	0	87	0	36	09	76	0	87	0
10	51	1	51	1	-	10	51	1	51	1
11	15	0	28	0	1	11	15	0	28	0
12	22	0	-	0	22	12	22	0	-	0
13	72	35	89	35	39	13	72	35	89	35
14	34	1	35	2	33	14	34	1	35	2
15	33	25	73	30	9	15	33	25	73	30
16	40	3	50	4	35	16	40	3	50	4
17	67	5	67	6	-	17	67	5	67	6
18	33	2	56	2	8	18	33	2	56	2

*BAS* basal, *AHI* apnea/hypopnea index, *MIN* minimum, *SUP* supine, *NSUP* no supine, *PRE* preoperative, *POST* postoperative, *PSO*_*2*_ peripheral oxygen saturation

In addition, mean, median, standard SD and interquartile range (IQR) of the tooth movements are presented in Table 4.

**Table 4:** Mean, median, standard deviation and interquartile range of the tooth movements

Statistical analysis	ADV-SCI (mm)	VAM-SCI (mm)	VPM-1.6 (mm)	VPM-2.6 (mm)	ADV-Pg (mm)
Valid	18	18	18	18	18
Lost	0	0	0	0	0
Mean	10.40	-2.11	1.61	1.71	14.47
Median	10.15	-2.00	1.50	1.60	13.50
SD	0.49	1.42	1.33	1.16	2.71
IQR	1.00	1.13	1.95	1.78	3.60
Percentages					
0%	9.7	-7.0	-1.0	0.0	11.3
25%	10.00	-2.13	0.98	1.00	12.68
50%	10.15	-2.00	1.50	1.60	13.50
75%	11.00	-1.00	2.93	2.78	16.28
100%	11.00	-1.0	4.1	3.9	22.3
K-S *p*	0.000	0.000	0.102	0.146	0.013

*ADV* advancement, *K-S-p* test Kolmogorov- Smirnov, *SCI* superior central incisor, *VAM* vertical anterior movement, *VPM* vertical posterior movement, *Pg* pogonion, *SD* standard deviation, *IQR* interquartile range

## Discussion

The results of this study reject the null hypothesis stating that there would be no changes in the upper airway volume after performing bimaxillary advancement surgery. The findings of the present study present a reduce of the upper airway volume after surgery, of 5144 mm^3^ in the right sinus cavity, 4621mm^3^ in the left and 8600mm^3^in the nasal cavities and maxillary sinuses complex. The asymmetric volume change in the maxillary sinus after bimaxillary advancement surgery might be due to differences in the preoperative volumes of both maxillary sinuses, or even to the midline correction performed to improve the upper dental midline of the patients with facial asymmetry.

It is important to take into consideration before making the decision to perform surgery in a patient with OSA, that the outcome of the procedure depends on many factors, such as OSA severity, body mass index and airway anatomy [14].

Bimaxillary advancement is typically performed, maintaining the pre-existing dental relationship and consists of Le Fort 1 osteotomy for maxillary advancement, as well as bilateral sagittal split osteotomy for mandibular advancement [15]. As a result, surgical movements of the maxilla can affect the morphology of maxillary sinuses and surrounding structures such as the nasal cavities [16].

The most important goal that can be achieved for OSA patients is the relief of the symptoms, as this is usually the reason for the patients to seek treatment [17]. Despite that, exists a weak scientific co-relation between subjective perception of symptoms and objective measurements [18]. The results of the present study agree with previous ones concerning statistically significant volume reduction in the maxillary sinuses after Le Fort1 advancement [19–22]. This outcome can be explained from the fact that in order to move maxilla in three dimensions it is necessary to separate it from the midface. Consequently, the maxillary sinus is also separated from the surrounding bone and the sinus mucosa is thickened [19]. Bone grafts may also be involved and contribute to the thickening [20] but there is no direct relationship between the volume decrease and the amount of maxillary advancement [19]. Nocini et al. [17], observed that as the maxilla is moved anteriorly, there is a thickening in the posterior maxillary sinus wall. An additional etiology could be that the inflammatory changes produced after surgery, provoke a reduction in the maxillary sinuses volume [21]. The clinical implications after maxillary sinus reduction remain unknown [18], although these changes don´t seem to negatively affect the airway [20, 23].

Nasal airway was also found to be reduced in volume [23]. The study of Erbe et al. [24] that measured nasal changes after Le Fort 1, confirmed through acoustic rhinometry that although nasal dimensions were decreased, airway resistance was not influenced. In addition, Spalding et al. [25], did not found consistent association between the amount or direction of maxillary surgical movement and nasal respiration. Ghoreishian and Gheisari [26] found that impaction and advancement of the maxilla can improve nasal respiratory function but impactation and setback would have the opposite result. Finally, the study Faur et al. [27] that evaluated rhinosinusal volume using CBCT, after LeFort 1 on virtual models, found that rhinosinusal complex and maxillary sinus volume was decreased in all patients postoperatively, but nasal fossa volume was decreased only in Class II malocclusion patients and increased in Class III patients. This result suggests that the direction of the sagittal movement of the maxilla plays an important role in nasal fossa volume while it does not directly affect the maxillary sinus volume. Surgical assisted rapid maxillaryexpansion (SARME) has also been proved to be efficient for reducing airway resistance and improve OSA parameters [28].

After treatment the patients described in the clinical report fewer symptoms such as snoring, daytime sleepiness and better function at workplace [28]. However, OSA is a chronic disease and requires long term follow up [2]. Bimaxillary advancement surgery is a common treatment that provides improvement of quality of life and reduction of the clinical symptoms, but it does not cure the syndrome [3]. Skeletal stability is achieved over time, but results are not well established concerning soft tissue stability and symptomatology [1]. More extended monitoring is needed in order to obtain solid results concerning long term response of patients suffering from OSA after bimaxillary advancement surgery.

## Conclusion

In conclusion, the results showed that bimaxillary advancement surgery reduces the maxillary sinus volume as well as, the nasal cavity volume.

## Data Availability

The datasets used and/or analysed during the current study are available from the corresponding author on reasonable request.
